# Role of Vitamin C in Selected Malignant Neoplasms in Women

**DOI:** 10.3390/nu14040882

**Published:** 2022-02-19

**Authors:** Anna Markowska, Michał Antoszczak, Janina Markowska, Adam Huczyński

**Affiliations:** 1 Department of Perinatology and Women’s Health, Poznań University of Medical Sciences, 60-535 Poznań, Poland; annamarkowska@vp.pl; 2 Department of Medical Chemistry, Faculty of Chemistry, Adam Mickiewicz University, 61-614 Poznań, Poland; michant@amu.edu.pl; 3 Department of Oncology, Gynecological Oncology, Poznań University of Medical Sciences, 60-569 Poznań, Poland; jmarkmed@poczta.onet.pl

**Keywords:** vitamin C, l-ascorbic acid, breast cancer, cervical cancer, endometrial cancer, ovarian cancer

## Abstract

Since the first reports describing the anti-cancer properties of vitamin C published several decades ago, its actual effectiveness in fighting cancer has been under investigation and widely discussed. Some scientific reports indicate that vitamin C in high concentrations can contribute to effective and selective destruction of cancer cells. Furthermore, preclinical and clinical studies have shown that relatively high doses of vitamin C administered intravenously in ‘pharmacological concentrations’ may not only be well-tolerated, but significantly improve patients’ quality of life. This seems to be particularly important, especially for terminal cancer patients. However, the relatively high frequency of vitamin C use by cancer patients means that the potential clinical benefits may not be obvious. For this reason, in this review article, we focus on the articles published mainly in the last two decades, describing possible beneficial effects of vitamin C in preventing and treating selected malignant neoplasms in women, including breast, cervical, endometrial, and ovarian cancer. According to the reviewed studies, vitamin C use may contribute to an improvement of the overall quality of life of patients, among others, by reducing chemotherapy-related side effects. Nevertheless, new clinical trials are needed to collect stronger evidence of the role of this nutrient in supportive cancer treatment.

## 1. Introduction

**Figure 1:**
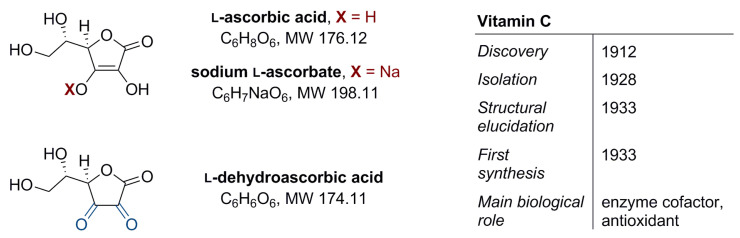
Structure of vitamin C (l-ascorbic acid, ascorbate), its sodium salt (sodium l-ascorbate), and oxidized form (l-dehydroascorbic acid) plus additional information on vitamin C [1].

Vitamin C (l-ascorbic acid, Figure 1) is water-soluble. It has a regulatory function in the metabolism of carbohydrates, proteins, and fats, and in the biosynthesis of selected hormones, enzymes, collagen, or iron absorption. It also participates in immunity building and shows antioxidant properties that indirectly protect cells against oxidative stress [1,2]. Vitamin C is exogenous and makes an essential component of the diet; its source is either food or appropriate supplementation. In food products vitamin C may occur in two forms, differing in chemical stability, in vivo half-life, and the transport mechanism [3]: (i) the reduced form–l-ascorbic acid (about 80–90%) and (ii) the oxidized form–l-dehydroascorbic acid (10–20%) (Figure 1) [4]. The recommended daily intake of vitamin C is in the range of 75–90 mg. This amount ensures an optimal plasma concentration of 30–90 µM [2,5].

Based on the role of vitamin C in the body, William J. McCormick hypothesized in the 1950s that cancer metastasis may be closely related to limitations in collagen formation and the degeneration of connective tissue resulting from deficiencies of this vitamin [6]. In the 1970s, Ewan Cameron et al. [7,8,9,10,11] described the effects of vitamin C use among cancer patients. They observed that ascorbate led to tumor growth inhibition by decreasing hyaluronidase activity, strengthening the extracellular matrix, reducing tissue damage, inhibiting tumor cell proliferation, and hindering metastasis [7]. In addition, a case report of 50 people with advanced cancer (published in 1974) indicated that vitamin C was beneficial in some cases [8]. A therapeutic effect of treatment with high doses of vitamin C was also observed in two other retrospective studies [10,11]. In both studies, high doses of ascorbate were administered intravenously and then orally to cancer patients [10,11]. Patients showed an improvement in the symptoms of the disease and a longer, up to four times, average survival time compared to the control group [10,11]. An independent clinical trial reported in Japan in 1982 showed a similar result [12]. Unlike the above-mentioned studies, the randomized, double-blind placebo-controlled trials published in 1979 and 1985, led by Charles Moertel, did not show any evidence on the effectiveness of a high dose vitamin C use in patients with cancer [13,14]. A more rigorous study by the Mayo Clinic has reduced enthusiasm for the potential use of vitamin C as a cancer-fighting agent [13,14].

However, there are at least two key methodical differences between these studies: (i) in the trials driven by Moertel, the administration of ascorbate was abruptly stopped and further switched to classical chemotherapy when the patients developed signs of cancer progression; (ii) Cameron and Pauling administered vitamin C both intravenously and orally, while in the studies of the Mayo Clinic, cancer patients received orally 10 g of ascorbate per day. This difference in the two dosage routes was crucial, but in none of the studies mentioned above were the plasma vitamin C concentrations measured, which may be fundamental in explaining its anti-cancer potential. Moreover, bearing in mind that in [13,14] the patients with cancer were administered vitamin C orally only, the results of these studies should not disqualify the potential anti-cancer efficacy of high vitamin C concentrations produced by its intravenous administration. 

### 1.1. Oral Versus Intravenous Delivery of Vitamin C

Given the pleiotropic effects of vitamin C, it was predicted that optimization of its concentrations should benefit cancer patients. Physiological analyses have shown that vitamin C pharmacokinetics significantly depended on the method of administration. Padayatty et al. [15] have observed that intravenous administration of vitamin C resulted in plasma concentration that was approximately 100 times higher (~15 mM) compared to that after oral administration (~100–200 µM). Another phase I clinical trial has shown that ascorbate can safely reach 25–30 mM after an intravenous infusion of 100 g of vitamin C [16]; notably, the plasma concentration of ~10 mM was maintained for a minimum of four hours which would be sufficient to kill cancer cells. Although the studies on a mouse model indicated that oral vitamin C supplementation may impair tumor growth and increase the rejection rate of implanted cancer cells [17,18], high concentrations of ascorbate were successfully used to selectively destroy human cancer cell lines, which has also been experimentally confirmed on animal cancer cell models (Table 1) [19]. 

Due to significant differences in pharmacokinetic properties, extensive research into the use of vitamin C in intravenous infusions (at ‘pharmacological concentrations’) as an alternative to oral administration (at ‘physiological concentrations’) has been performed (Table 2) [3,5,19,36,37,38,39,40]. For example, the intravenous infusion of ascorbate at a dosage of 25 g daily, increased gradually to 75 g daily, started soon after completion of the standard therapy, might help to prevent the recurrence of stage IV ovarian cancer, without the negative chemotherapy-related side effects [41]. The intravenous route of vitamin C administration appears to be of particular interest, especially in the light of the results of the studies by Long et al. [42] on the effect of dietary vitamin C oral intake on the risk of ovarian cancer development. The above authors searched electronic databases analyzing 16 studies involving 439,741 participants, including 4553 patients with ovarian cancer [42]. Dietary vitamin C consumption had no significant effect on the risk of ovarian cancer development (RR 0.95, 95% CI 0.81–1.11) [42]. However, this analysis had some limitations; almost all studies were performed in one geographical region (North America), and factors such as BMI (body mass index) and the duration of oral contraceptive use were not taken into account [42]. In the authors’ opinion, for the correct validation of the obtained results, cohort studies on a larger number of patients are necessary [42]. Vitamin C also contributed to improving treatment outcomes among patients with breast cancer [43,44], but these studies used survey data to ascertain vitamin C status, not actual measurements.

Importantly, it has been noted that patients with malignant neoplasms usually had lower mean plasma concentrations of vitamin C compared to healthy subjects [50,51,52,53,54,55,56]. These patients, in many cases, were diagnosed with hypovitaminosis (<23 μM) or complete deficiency (<11 μM) of this nutrient [57,58,59,60,61,62,63]. Vitamin C deficiency was also correlated with an increased risk of death from the disease [59]. Aune et al. [64] presented the meta-analysis results of five studies involving 45,758 participants; a 50 μM increase in vitamin C concentrations was associated with an approximately 26% lower risk of cancer in various body locations. In contrast, a meta-analysis of 52,018 Europeans on genetic variants related to plasma vitamin C concentrations and potential cancer development did not indicate a causal relationship between circulating vitamin C concentrations and any of the five most common cancers, including breast cancer [65].

### 1.2. Anti-Cancer Mechanism of Action of Vitamin C

**Figure 2:**
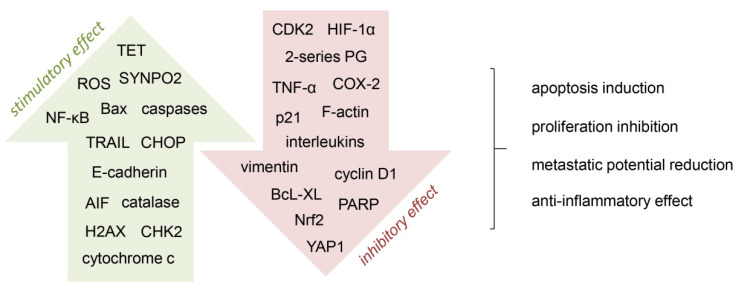
Mechanisms involved in the anti-cancer activity of vitamin C. A number of cell processes are targeted by vitamin C, by stimulating (green arrow) or inhibiting (red arrow) different pathways. AIF, apoptosis-inducing factor; CDK2, cyclin-dependent kinase 2; CHK2, checkpoint kinase 2; CHOP, C/EBP homologous protein; COX-2, cyclooxygenase-2; H2AX, histone 2AX; HIF-1α, hypoxia-induced factor 1α; NF-κB, nuclear factor-κB; Nrf2, nuclear factor erythroid 2-related factor 2; PARP, poly(ADP-ribose) polymerase; PG, prostaglandins; ROS, reactive oxygen species; SYNPO2, synaptopodin 2; TET, ten-eleven translocation; TRAIL, tumor necrosis factor (TNF)-related apoptosis-inducing ligand; YAP1, yes-associated protein 1 [21,26,27,28,32,38,68,69,70].

The anti-cancer effect of vitamin C is based, among others, on cancer cell programmed death (apoptosis) induction, proliferation inhibition, and reduction of metastasis potential (Figure 2). Vitamin C may be involved in the regulation of microRNA and numerous oncogenic signaling pathways, such as JAK-STAT (Janus kinase/signal transducer and activator of transcription), TGF (transforming growth factor)/SMAD, and TRAIL (tumor necrosis factor (TNF)-related apoptosis-inducing ligand) [38,66]. Tumor hypoxia leads to an aggressive cancer phenotype which facilitates invasion and significantly increases the risk of metastasis; vitamin C acts as a cofactor of selected enzymes (hydrolases), inhibiting the activity of the hypoxia-induced factor 1α (HIF-1α) [20,38,67]. Other studies suggest that vitamin C may be involved in restoring the activity of TET (ten-eleven translocation) enzymes, leading to epigenetic reprogramming and, thus, influencing the regulation of cancer cell growth [5,68]. In vitro and in vivo tests have shown that vitamin C supported cancer immunotherapy in various locations (breast, colorectal, melanoma, and pancreatic murine tumors); it increased the cytotoxicity of adoptive CD8 T lymphocytes and significantly enhanced the immunological activity of checkpoints in the tumor microenvironment [39]. Intravenous administration of ‘pharmacological doses’ of vitamin C in combination with selected chemotherapeutic agents may contribute to the selective destruction of cancer stem cells (CSCs), whose presence is associated with drug resistance and cancer relapse [40]. In vitro, the effects of vitamin C on breast cancer cells by promoting apoptosis through various mechanisms have been observed [21,69]. One of these mechanisms was to induce apoptosis through AIF (apoptosis-inducing factor), which in normal cells is retained in the mitochondria, and under the influence of vitamin C, it was translocated to the nucleus, activating caspase-independent programmed cell death [21]. According to Sant et al. [69] vitamin C induced apoptosis by increasing TRAIL expression, which, in turn, activated pro-apoptotic Bax and caspases, as well as decreased the anti-apoptotic regulator BcL-XL. Relatively high doses of vitamin C (2 mM) inhibited migration and invasion of breast cancer cell lines by suppressing epithelial-mesenchymal transition (EMT) [26]. In the case of triple negative breast cancer, the plasma concentration of vitamin C achieved after oral administration (100 µM) inhibited the metastatic potential of cancer cells, influencing the expression of YAP1 (yes-associated protein 1) and synaptopodin 2, both genes in the Hippo pathway [27], while at ‘pharmacological concentrations’ (≥20 mM) it also influenced CSCs [28]. 

Although the in vitro experiments provided the necessary information on the potential mechanisms of anti-cancer activity of vitamin C, and the results of preclinical studies showed promising efficacy of the intravenous route of its administration, the conducted clinical trials gave results mainly of phase I and II studies (Table 3) [36,61,71,72,73]. Moreover, the results of clinical trials are inconclusive and have not delivered solid evidence of clinical efficacy. Nevertheless, high doses of vitamin C are administered intravenously by complementary and alternative medicine practitioners as an additional treatment option for patients [74]. The relatively high popularity of vitamin C use among cancer patients, often outside the hospital system, means that the effects of this therapy may not be fully appreciated. Therefore, in this article, we pay special attention to the possible beneficial effects of vitamin C in the treatment of selected malignant neoplasms in women, including breast, cervical, endometrial, and ovarian cancer. The Google Scholar and PubMed databases were perused for eligible evidence published mainly in the last 20 years, including in vitro tests, observational studies, clinical trials, and meta-analyses.

## 2. Breast Cancer

Breast cancer (BC) is the most commonly diagnosed type of cancer among women and has multifactorial pathology, including gene mutations, hormonal disorders, and lifestyle. Oncological patients have a reduced vitamin C status, which may be related to increased metabolic turnover, as a result of oxidative and inflammatory aspects of the disease process [75]. As a result, it is hypothesized that cancer patients have a greater need for vitamin C. Intravenous administration of vitamin C to BC patients results in lower circulating concentrations of vitamin C compared to administration of the same amount to healthy controls [76], indicating a higher demand for this vitamin in cancer patients. Interestingly, patients with higher-stage BC (and cervical cancer) had significantly lower concentrations of vitamin C than those with earlier stages of the disease [77,78]. Despite the promising results of in vitro tests, meta-analyses, and observational studies, the actual efficacy of vitamin C in intravenous infusions during BC therapy remains debatable [20,21,22,40,68,69,79].

### 2.1. In Vitro and In Vivo Activity 

Studies by Lee et al. [20] on a variety of BC cell lines have shown that a high dose of vitamin C (≥10 mM) induces an apoptotic effect in cancer cells; the same effect was observed both with the use of vitamin C alone and in combination with drugs commonly used in the treatment of BC, such as tamoxifen, fulvestrant, and trastuzumab [20]. Moreover, a high dose of vitamin C had an antiproliferative effect on cell lines resistant to the action of oncological drugs (tamoxifen, doxorubicin, and docetaxel) [20]. The authors of [20] believe that the use of vitamin C in high doses in combination with conventional anticancer agents may therefore bring therapeutic benefits. Similarly, in vitro studies have shown a beneficial effect of vitamin C in combination with mitoxantrone (an analog of anthracycline antibiotics) used in the treatment of many malignant neoplasms, including BC [22]. Vitamin C, administered in combination with this drug, resulted in a higher level of cytotoxicity in neoplastic cells compared to the therapy with the use of the cytostatics alone [22]. This indicates the possibility of reducing the mitoxantrone dose which is important due to a number of undesirable side effects such as cardiotoxicity, leukopenia, and myelosuppression [22]. Vitamin C in combination with auranofin, an oral chrysotherapeutic agent used in the treatment of rheumatoid arthritis, has shown promising results in the control of triple-negative BC in vitro, but also in in vivo tests performed on mice-bearing MDA-MB-231 xenografts [23]. As far as triple-negative BC is concerned, the use of vitamin C sensitized neoplastic cells to bromodomain and extra-terminal (BET) protein inhibitors [24]. El Banna et al. [25] have found that ‘pharmacological doses’ of vitamin C (10 mM) were cytotoxic against most of BC cell lines tested without severely affecting normal cells, suggesting that rational combinations of treatments based on vitamin C could represent new therapeutic options for triple-negative BC. To sum up, vitamin C at mM concentrations was cytotoxic for a series of human cancer cell lines. At plasma concentrations which may be clinically reachable by intravenous administration, this nutrient induced death in most of cancer cell lines studied, and had no toxic effect on normal cells, demonstrating its promising biological activity profile.

On the other hand, using the MCF-7 cell line, an adverse effect of vitamin C on treatment with tamoxifen, a selective estrogen receptor modulator commonly used in women with BC and positive estrogen receptor (ER) expression, was demonstrated [80]. The results showed that vitamin C antagonizes the cytotoxic effects of tamoxifen, thereby protecting cancer cells from lipid peroxidation [80]. According to the authors of this study, supplementation with vitamin C during tamoxifen-based therapy requires validation in other therapeutic models and/or in clinical trials [80].

### 2.2. Effects in Cancer Patients

A very interesting direction of research is validation whether lifelong exposure to ascorbate has a protective effect on cancer. The analysis based on the Swedish Mammography Database questionnaires, including 3405 women (median follow-up 7–8 years), also showed a beneficial effect of vitamin C, reducing the risk of death from BC, especially in the group of women around 65 years of age [43]. Looking closer at the results, the authors found that dietary intake of ascorbate before BC diagnosis was connected with BC-specific survival; this correlation was particularly common among women aged ≥65 [43]. Furthermore, a harmful effect of post-diagnosis supplementation with vitamin C (∼1.0 g) was not found [43]. In their meta-analysis based on electronic databases of the course of the disease in over 17,000 cases of BC, Harris et al. [44] also showed that supplementation with vitamin C reduces the overall risk of death in BC patients, as well as the risk of death specific to cancer itself (RR 0.85, 95% CI 0.72–0.91 and RR 0.85, 95% CI 0.74–0.99, respectively). Post-diagnosis supplementation with ascorbate did not have a negative impact on BC survival and was statistically significantly correlated with a reduced risk of mortality, while dietary vitamin C intake was linked with a reduced risk of total mortality as well as BC-specific mortality [44]. Using a retrospective analysis of tumor tissue, Campbell et al. [81] have found a direct association between intracellular vitamin C contents and activation of the HIF-1 pathway, as well as patient survival in BC; the results suggested that optimization of tumor ascorbate concentrations might modulate the hypoxic response, with potential clinical benefits. Zhang et al. [82] claim that the relationship between vitamin C intake and BC is unclear; based on the analysis of electronic databases of 54 studies on the risk of BC development and 15 studies on the survival of cancer patients, they have found that vitamin C, contained in the diet (but not in supplements), reduces the risk of developing the disease (RR 0.89, 95% CI 0.82–0.96) and lowers mortality (RR 0.82, 95% CI 0.74–0.91). The authors suggest that the use of vitamin C supplements is of little importance in the prevention of BC [82]. Less promising research results have been obtained by the UK Dietary Cohort Consortium; based on diary recordings from 707 BC patients and 2144 controls, no significant association was found between the incidence of BC and diet or total vitamin C intake [83].

BC patients, when undergoing radiation therapy, very often experience some complications, including pulmonary fibrosis and radiation pneumonia, which are accompanied by an increase in the inflammatory level, which acts as a prognostic factor that may lead to increased mortality among cancer patients [84]. Thus, to support the positive role of vitamin C in women, Park et al. [45] have studied the effect of intravenous vitamin C administration on inflammation in radiotherapy undergoing postoperative BC patients; 354 patients were assigned to two groups: (i) patients treated with radiotherapy assisted by intravenous administration of vitamin C (for at least four weeks) and (ii) patients undergoing radiation therapy only [45]. Neutrophil-lymphocyte ratio (NLR) assumed as an indicator of inflammation and predictive survival factor was measured in all subjects [45]. It was found that increased NLR was associated with a higher mortality, which was reduced in the group of patients undergoing radiotherapy and receiving high doses of vitamin C [45]. This result may confirm the anti-inflammatory effect of vitamin C by modulating cytokines IL(interleukin)-1α, IL-2, IL-6, IL-8 and TNF-α (Figure 2) [85], leading to the restoration of physiological vitamin C concentrations and improvement of patient quality of life [68]. 

**Figure 3:**
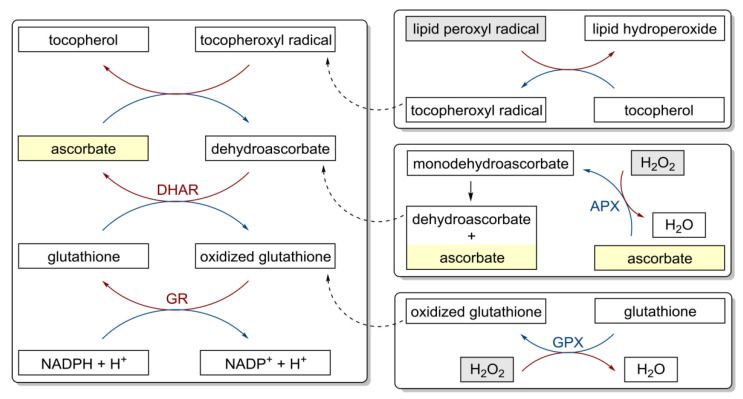
Antioxidant properties of vitamin C and redox cycling antioxidants. APX, ascorbate peroxidase; DHAR, semidehydroascorbate reductase; GPX, glutathione peroxidase; GR, glutathione reductase [86].

Although the adjuvant treatment of BC patients with intravenous vitamin C was a well-tolerated optimization of standard treatment regimens, reducing side effects [46], given the antioxidant properties of vitamin C (Figure 3) [86], many clinicians believe that concomitant use of intravenous vitamin C should be avoided in all standard chemotherapy regimens. 

However, chemotherapeutic agents act by various mechanisms, only some follow strictly oxidative mechanisms [87]. Moreover, vitamin C acts as an antioxidant at ‘physiological concentrations’, but it can also show pro-oxidative properties when used in much higher concentrations (>1 mM) [29]. As vitamin C has a relatively short half-life (<2 h) due to rapid renal clearance, intravenous vitamin C infusions are usually given to patients the day before or after administration of standard chemotherapy [73,88]. It has been noticed that the application of standard treatment regimens using various cytostatics (5-fluorouracil, cisplatin, interleukin-2, and nilotinib) can significantly reduce vitamin C concentrations in patients and, in some cases, lead to symptoms of scurvy [58,89,90,91,92]. Of note, discontinuation of chemotherapy or the use of supplementation eliminated the symptoms of this vitamin deficiency [58,91,92]. On the other hand, supplementation of vitamin C (Celin 500 mg) and vitamin E (Evion 400 mg) combined with tamoxifen (10 mg, twice daily) was effective in reducing tamoxifen-induced hypertriglyceridemia among postmenopausal BC patients [47]. Other studies on 60 postmenopausal women with BC have also shown that concomitant administration of vitamin C is beneficial in patients treated with tamoxifen [48]. 

## 3. Cervical Cancer

Cervical cancer (CC) is the most frequently diagnosed malignant neoplasm occurring in low or middle-income countries. This type of cancer is the result of a long process of changes in the normal cervical epithelium following persistent HPV (human papillomavirus) infection [30,93,94]. Moreover, it has been shown that the concentration of vitamin C may be significantly reduced in patients with CC compared to healthy subjects [95,96,97,98]. The lower the concentration of vitamin C in the serum, the higher the advancement of the neoplastic disease [77].

### 3.1. In Vitro Activity 

A study by Sindhwani et al. [31] on the CC (HeLa) cell lines showed that vitamin C caused a reduction in their viability. This is also confirmed by the in vitro studies carried out by Wu et al. [32]. They assessed the effect of ascorbic acid not only on the viability of neoplastic cells, but also on proteins related to the cell cycle (p53, p21, cyclin D1) and the value of the antioxidant transcription factor (Nrf2, nuclear factor erythroid 2-related factor 2), which were lowered (Figure 2) [32]. Vitamin C at relatively high concentrations (7 mM and 10 mM) induced HeLa cell death via the external and internal apoptotic pathway [29]. Additionally, vitamin C in ‘pharmacological doses’ (1–10 mM) increased the susceptibility of HeLa cells to the action of cisplatin and doxorubicin [32]. The authors suggest that the potential synergistic use of vitamin C in combination with cytostatics may increase the effectiveness of anti-cancer therapies [32]. Leekha et al. [30] have confirmed that vitamin C enhances the effect of cisplatin used in the treatment of CC without affecting normal cells, which was associated with p53 overexpression and the production of toxic hydrogen peroxide in cancer cells. The results of other studies have shown that vitamin C enhances the chemotherapeutic response of HeLa CC cells by stabilizing p53 [33]. In our opinion, intensive in vitro studies should be focused on the possible synergistic interactions of vitamin C with commonly used anti-cancer drugs to ensure the effectiveness and tumor-specificity of such a treatment strategy.

### 3.2. Effects in Cancer Patients

Observational studies have shown that antioxidants may inhibit the development of cervical diseases associated with HPV infection. A case-control study conducted in South Korea, involving 144 cases of invasive CC and 288 women in the control group, has shown that the consumption of antioxidant vitamins, including vitamin C, reduces the risk of developing CC (OR 0.35, 95% CI 0.19–0.66) [99]. Similarly, based on a case-control study in China (458 women with invasive cancer and 742 as the control group), it has been found that vitamin C intake may reduce the risk of developing invasive CC (*p* < 0.001) [100]. Ono et al. [94] claim that further in vitro and in vivo studies are necessary to explain such relationships. On the basis of the analysis of electronic databases, Cao et al. [101] assessed the relationship between vitamin C intake and the risk of CC. This analysis included one prospective cohort study and 11 case-control studies [101]. Vitamin C consumption was associated with a lower risk of developing CC; overall, meta-analysis showed a reduction in the development of this type of neoplasm (OR 0.58, 95% CI 0.44–0.75, *p* < 0.001) [101]. However, a lowered consumption of vitamin C increased the risk of developing CC, and this effect was dependent on the dose used [101]. The authors believe that these results may support the further validation of randomized controlled trials [101]. Nevertheless, more advanced studies are greatly needed to find and validate the positive drug-drug interactions between vitamin C and commonly used anti-cancer chemotherapeutics.

## 4. Endometrial Cancer

Endometrial cancer (EC) is the most common malignant neoplasm of the female reproductive system, especially in economically developed countries. The factors increasing the risk of developing this type of cancer in women, both before and after menopause, are hypertension, diabetes, obesity, and hormonal imbalance [102]. Although increasing body of evidence has supported the positive effects of the use of vitamin C in EC patients, the numbers of in vitro and animal studies are very limited; in our opinion, this issue should be intensively studied and completed in the coming years. 

### Effects in Cancer Patients

Bandera et al. [103], having performed a systematic review of literature and a meta-analysis, were the first to publish the results of research on the influence of antioxidant vitamins, including vitamin C, on the development of EC. The reduced risk of developing EC (one cohort and 10 case-control studies) was related to the high vitamin C content in food (50 mg vitamin C per 1000 calories consumed; OR 0.85, 95% CI 0.73–0.98, *p* < 0.01) [103]. Higher-grade EC contained proportionally less vitamin C than lower-grade tumors [104]. Low concentrations of ascorbate have been found especially in larger tumor size samples; they were also associated with increased levels of VEGF (vascular endothelial growth factor) and GLUT-1 (glucose transporter 1) [104]. Compared to non-neoplastic tissue, endometrial neoplasms showed a lower concentration of ascorbate, which, in turn, was associated with HIF-1α activation [103]. The highest levels of HIF-1α were observed in high-grade endometrial neoplasms [104]. The correlation between low ascorbate concentrations and HIF-1α was statistically significant (*p* = 0.007) [104]. According to the authors, low vitamin C concentrations could be associated with HIF-1α activation and the aggressive phenotype of EC [104]. Yasin et al. [105] found that diet and lifestyle are also among the factors influencing EC; this applies to both the prevention and promotion of this type of cancer development. Although some studies suggest a positive effect of vitamin C supplementation in reducing the risk of EC [106], the results obtained by other authors indicate that there is no clear relationship between dietary vitamin C intake or the use of appropriate vitamin C supplements and the risk of EC [107,108,109,110]. Moreover, some efforts should focus on exploring novel drug–drug combinations with optimal dose of vitamin C, which seem to be of great value and have the potential to yield an improvement in efficacy and safety profiles in possible combination treatment.

## 5. Ovarian Cancer

Ovarian cancer (OC) is the leading cause of death among all malignant gynecological neoplasms. The majority of cases, about 70%, are, unfortunately, diagnosed in the advanced stages of the disease. Despite a positive response to primary treatment (chemotherapy or surgery) and the use of targeted therapies, most cancer patients develop refractory, fatal relapse [111,112]. Low concentrations of vitamin C have been found in the plasma of patients with OC [113]. As in the case of the previously discussed types of neoplasms, and in the case of OC, the results of studies (especially observational) on the potential use of vitamin C as an alternative method in treating this type of cancer are ambiguous.

### 5.1. In Vitro and In Vivo Activity 

Vitamin C has been shown to have a toxic effect on OC cells (OVCAR-3) [70]. At the ‘pharmacological dose’ (1 mM), vitamin C decreased cell proliferation, mainly through the inhibitory effect on cyclin-dependent kinase 2 (CDK2) and a reduction of PARP (poly(ADP-ribose) polymerase) expression (Figure 2) [70]. However, it had no adverse impact on non-neoplastic cells [70]. The authors of the study suggest that vitamin C could be an adjuvant in the treatment of OC [70]. Furthermore, in vitro studies have shown that vitamin C can reduce the proliferation of ID8 OC cells inducing apoptosis and cell cycle arrest [35]. Moreover, vitamin C lowered the level of macrophages in the tumor, and reduced the EMT and the formation of spheroids [35]. These mechanisms reduced tumor invasiveness and potential for metastasis of the tumor to the peritoneum [35].

Antioxidants added to first-line chemotherapy may improve treatment effectiveness. In this context, Ma et al. [34] have shown in an in vivo model that vitamin C administered parenterally to mice implanted with cancer cells induces the death of these cells, especially when vitamin C is used in combination with carboplatin and paclitaxel–commonly used oncological drugs in the fight against OC; the observed synergistic effect resulted in greater eradication of tumor cells compared to the effects of the treatment with each drug alone. 

### 5.2. Effects in Cancer Patients

While some studies indicated that vitamin C supplementation may be associated with a reduced risk of OC [114], the results of other studies have not provided such clear conclusions [115,116,117]; some have even delivered contradictory evidence [118,119,120,121]. On the basis of electronic databases, including the above-cited study by Ma et al. [34] on OC, Nauman et al. [122] have reviewed the effects of intravenous vitamin C administration in people with malignant tumors in various body locations. Analysis of the data gathered from 23 studies involving 385 patients, has provided grounds for these authors to recommend intravenous administration of vitamin C at a dose of 1 g kg^−1^ of body weight at least twice a week for a period of 2–3 months to assess the safety and possible effectiveness of treatment [122]. The results of preclinical studies indicate that a single infusion of vitamin C is not as effective as multiple infusions, and a higher frequency of administration appears to be more beneficial [123,124].

The authors of [34] also described the consequences of vitamin C administration in combination with standard chemotherapy in patients with stage III/IV disease. A relatively small number of patients (25 women) were randomly assigned to two groups [34]. The first of these groups (13 women) received standard chemotherapy (carboplatin and paclitaxel) for one year in combination with intravenous administration of vitamin C (75 g or 100 g per infusion, depending on peak plasma concentration of each individual, twice a week), and the second group (12 women) was treated only with standard chemotherapy [34]. In the ascorbate-treated group, progression-free survival (PFS) was 25.5 months, while in the group with chemotherapy alone, it was approximately 16.8 months [34]. There were fewer side effects in the group of patients receiving vitamin C infusions [34]. The anti-cancer activity of the combination of chemotherapy with vitamin C was mediated by AMPK (AMP-activated protein kinase) activation, which inhibited the signaling of the mTOR (mammalian target of rapamycin kinase) pathway closely related to cancer progression [34]. In these authors’ opinion, parenteral administration of vitamin C may increase the sensitivity of neoplastic cells to the applied standard chemotherapy; therefore, it seems justified to conduct further clinical trials in this area [34]. In another study, vitamin C was administered first orally, then parenterally (60 g twice weekly), and used as an adjunctive option to the first-line chemotherapy, which resulted in the inhibition of the progress of the neoplastic disease in two cases of OC [49]. 

## 6. Conclusions

Vitamin C is essential for the proper functioning of the body. In addition to its antioxidant activity, vitamin C plays an important regulatory role, it is involved in the biosynthesis of hormones and enzymes, and the process of iron absorption. In addition, an increasing number of reports indicate the potential role of vitamin C in the prevention of selected malignant neoplasms in women, including breast, cervical, endometrial, and ovarian cancer. The most widely accepted anti-cancer mechanisms of vitamin C action include: indirect production of hydrogen peroxide, enzyme cofactor activities (collagen synthesis, HIF hypoxic response regulation, TET regulation), antioxidant, and anti-inflammatory properties. Moreover, preclinical and clinical studies indicate the ability of intravenous vitamin C to reduce the side effects of chemotherapeutic agents without compromising their efficacy, which contributes to improvement of the overall quality of life of cancer patients. Low cost and toxicity may also be important in the potential use of vitamin C as an alternative adjuvant in cancer therapy.

Vitamin C deficiency is common in patients with advanced cancer, very often leading to hypovitaminosis. The use of classic chemotherapy may also reduce the concentration of circulating vitamin C. At the same time, patients with low plasma vitamin C concentrations often live shorter lives compared to people with normal or higher vitamin C concentrations. Nevertheless, the findings of the research into the anti-cancer effects of vitamin C are inconclusive. While some studies show the promising effect of vitamin C on cancer cells, others provide quite the opposite evidence. Essential limitations of the studies conducted with the use of vitamin C should also be considered. For instance, with respect to the possible autoxidation of vitamin C in cell culture media due to the presence of trace metals, the results from in vitro tests may differ from those obtained in cancer patients. For in vivo experiments, the studies using immunodeficient mice for the evaluation of the anti-cancer effects of vitamin C may provide information on its direct effects on tumors. However, it should be noted that the studies on mice that are able to synthesize their own vitamin C are different from those performed on Gulo-knockout mice or humans. Finally, questionnaires and surveys are not the same as actual measurements. The fact that many studies conducted so far are of low quality is not without significance. Therefore, in order to validate the obtained results, it is necessary to conduct large-scale, randomized clinical trials, which would be preceded by a carefully defined clinical design and selection of endpoints for evaluation, taking into account, for example, the dose, time, and route of vitamin C administration, or the type of cancer being treated. 

As cancer patients show reduced concentrations of vitamin C, the routine administration of this nutrient is not only warranted, but highly desirable. In the light of the results obtained so far, it seems that the most advantageous solution could be the use of vitamin C as a complementary agent supporting the action of commonly used oncological drugs, especially since the use of standard chemotherapy may have a negative impact on the concentration of this vitamin in the body. Vitamin C, when added to conventional chemotherapeutic drugs, may decrease the concentrations of these agents that produce cell killing, while possible synergy favors a higher ratio of ascorbate in the combination. Of note, it is widely accepted that the mM concentration of vitamin C needed to promote cytotoxic effects in cancer cells may be achieved only when this nutrient is administered intravenously. Importantly, such ‘pharmacological concentrations’ of vitamin C from intravenous dosing are well tolerated by patients. With respect to its low price, limited number of side effects, a clear-cut improvement of the quality of life of cancer patients and a promising anti-cancer activity, it seems there is a solid rationale for the possible introduction of vitamin C administration via intravenous infusions to the treatment of selected malignant neoplasms in women. Looking closer at the data collected and described in this review article, the chances of introducing vitamin C as a supportive agent in anti-cancer therapy appear to be the greatest for breast and ovarian cancer. However, it should be emphasized at this point that, pending the completion of clinical trials, it is premature to establish the role vitamin C may play in cancer treatment or secondary prevention.

## Figures and Tables

**Table 1:** In vitro (and animal) studies with vitamin C on cancer cell lines.

Cancer Type	Cancer Cell Line	Optimal Vitamin C Concentration	Combination Treatment	In Vivo	Reference
Breast cancer	MCF-7, MDA-MB-231, SK-BR-3	≥10 mM	eribulin mesylate, fulvestrant, tamoxifen, trastuzumab		[20]
	Hs578T, SK-BR-3	1.0 mM			[21]
	MCF-7, MDA-MB-231	1.0–1.5 mM	mitoxantrone		[22]
	MDA-MB-231	2.5 mM	auranofin	+	[23]
	MDA-MB-231	100 µM	BET inhibitors, especially JQ1	+	[24]
	HCC-1428, MDA-MB-134, MDA-MB-231, MDA-MB-415, MDA-MB-453, T47D	10 mM			[25]
	Bcap37, MDA-MB-453	2.0 mM		+	[26]
	BT-549, MDA-MB-231	100 µM		+	[27]
	MDA-MB-231	≥20 mM			[28]
Cervical cancer	HeLa	7.0–10 mM			[29]
	SiHa	100 µg mL^–1^	cisplatin		[30]
	HeLa	5.0–8.0 mM			[31]
	HeLa	1.0–10 mM	cisplatin, doxorubicin		[32]
	HeLa	1.0 µM	adriamycin, bleomycin, etoposide, cisplatin		[33]
Ovarian cancer	A2780, OVCAR3, OVCAR5, OVCAR8, OVCAR10, SHIN3, SKVO3	0.3–3.0 mM	carboplatin, paclitaxel	+	[34]
	ID8	1.5–2.0 mM		+	[35]

^+^ The results from studies on animal models are available.

**Table 2:** Observational studies on the use of vitamin C with chemo- or radiotherapy in selected malignant neoplasms in women.

Cancer Type	Vitamin C Administration	Dose of Vitamin C	Study Protocol	Reference
Breast cancer	intravenously	<1.0 g kg^−1^ or >1.0 g kg^−1^	Vitamin C was administered twice a week for at least four weeks during radiation therapy	[45]
	intravenously	7.5 g per infusion	Vitamin C was administered once a week during adjuvant therapy, for a minimum of four weeks; vitamin C was not administered on the days of chemo- and radiotherapy	[46]
	orally	Celin 500 mg	Vitamin C was administered along with vitamin E (Evion 400 mg) and tamoxifen (10 mg twice a day) for 45 or 90 days	[47]
	no data	no data	Cancer patients were treated with vitamin C after 45 or 90 days with tamoxifen	[48]
Ovarian cancer	intravenously	25–75 g daily	Vitamin C infusion progressively increased up to 75 g per day over a period of 28 days, then it was maintained two times a week for 12 months, and once a week for next six months. The treatment was further reduced to one dose every two weeks for another six months and finally to every three or four weeks until five years post-operation	[41]
	orally, then parenterally	Case 1: 9.0 g plus 15–60 g per infusion Case 2: 3.0 g plus 15–60 g per infusion	Case 1: Vitamin C infusions were given two times per week, after which the patient continued vitamin C infusions once per week Case 2: Vitamin C infusions for one week and then began twice weekly infusions, which continues to date 36 months post-diagnosis	[49]

**Table 3:** Ongoing and completed clinical trials with vitamin C in selected malignant neoplasms in women (ClinicalTrials.gov; online accessed on 14 January 2022).

Cancer Type	Title of Clinical Trial	Study Description	Status	Publications of Results	ClinicalTrials.gov (accessed on 14 January 2022) Identifier
Breast cancer	Effect of vitamin C and E in breast cancer patients undergoing chemotherapy	Determining effects of vitamin C (and vitamin E) use in combination with chemotherapeutic agents in breast cancer patients	Recruiting	No results posted	NCT04463459
	Intravenous ascorbic acid supplementation in neoadjuvant chemotherapy for breast cancer	Randomized (phase I/II) study of the effects of parenteral administration of vitamin C in addition to conventional cancer therapy in women with breast cancer	Unknown	No results posted	NCT03175341
Cervical (and ovarian) cancer	Safety of antioxidants during GYN cancer care	Pilot study (phase II) to assess the safety and efficacy of high doses of antioxidants, including vitamin C, in patients with gynecological cancer (cervical cancer, uterine cancer or ovarian cancer)	Completed	No results posted	NCT00284427
Ovarian cancer	Treatment of newly diagnosed ovarian cancer with antioxidants	Determining the possible benefits or harms of using antioxidant dietary supplements, including vitamin C, in combination with classic oncological drugs (paclitaxel, carboplatin) in the treatment of ovarian cancer	Completed	Administration of vitamin C together with chemotherapeutic agents (paclitaxel, carboplatin) reduced the toxicity associated with the use of oncology drugs in patients with ovarian cancer [34]	NCT00228319

## Data Availability

Not applicable.
